# 2,6-Dimethyl-4-oxo-3-oxatri­cyclo­[5.2.1.0^2,6^]decane-1-carboxamide

**DOI:** 10.1107/S1600536813017248

**Published:** 2013-06-29

**Authors:** Oleksandr Grytsai, Marian Gorichko

**Affiliations:** a National Taras Shevchenko University, Department of Chemistry, Volodymyrska str. 64, 01033 Kyiv, Ukraine

## Abstract

In the title compound, C_12_H_17_NO_3_, which was synthesized by Wagner–Meerwein rearrangement of the *N*-nitro­imine, the ring-junction C—C bond length is comparatively long [1.573 (2) Å] due to a steric repulsion between the methyl groups at these atoms, which also leads to an increase in the C—C—C angles along this C_4_ chain [118.10 (13) and 115.04 (15) °, respectively]. In the crystal, N—H⋯O—C and N—H⋯O=C hydrogen bonds are formed between the amide group and the two O-atom acceptors of the lactone group, forming a chain along [001].

## Related literature

For applications of nitro­imines and their derivatives in organic synthesis, see: Squire *et al.* (2002 ▶); Bulman Page *et al.* (2000 ▶); Lalk *et al.* (1999 ▶), as organocatalysts, see: Parrott, *et al.* (2008 ▶) and in medicinal chemistry, see: Ranise *et al.* (1990 ▶); Bondavalli *et al.* (1987 ▶). For bond angles in related structures, see: Noe *et al.* (1996 ▶); Knollmuller *et al.* (1998 ▶).
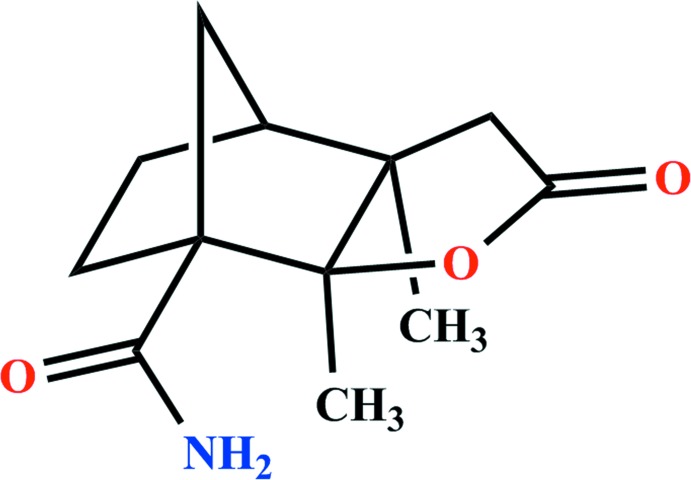


## Experimental

### 

#### Crystal data

C_12_H_17_NO_3_
*M* *_r_* = 223.27
Triclinic, 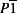

*a* = 7.0659 (3) Å
*b* = 7.8206 (3) Å
*c* = 10.4595 (3) Å
α = 79.667 (2)°
β = 80.471 (2)°
γ = 81.579 (2)°
*V* = 556.69 (4) Å^3^
*Z* = 2
Mo *K*α radiation
μ = 0.10 mm^−1^
*T* = 296 K
0.25 × 0.2 × 0.15 mm

#### Data collection

Bruker SMART APEXII CCD area-detector diffractometer
Absorption correction: multi-scan (*SADABS*; Bruker, 2008 ▶) *T*_min_ = 0.977, *T*_max_ = 0.986
8729 measured reflections
2405 independent reflections
1754 reflections with *I* > 2σ(*I*)
*R*_int_ = 0.037

#### Refinement

*R*[*F* ^2^ > 2σ(*F* ^2^)] = 0.044
*wR*(*F* ^2^) = 0.141
*S* = 0.92
2405 reflections
213 parameters
H atoms treated by a mixture of independent and constrained refinement
Δρ_max_ = 0.17 e Å^−3^
Δρ_min_ = −0.21 e Å^−3^

### 

Data collection: *APEX2* (Bruker, 2007 ▶); cell refinement: *SAINT* (Bruker, 2007 ▶); data reduction: *SAINT*; program(s) used to solve structure: *SHELXS97* (Sheldrick, 2008 ▶); program(s) used to refine structure: *SHELXL97* (Sheldrick, 2008 ▶); molecular graphics: *SHELXTL* (Sheldrick, 2008 ▶); software used to prepare material for publication: *SHELXTL*.

## Supplementary Material

Crystal structure: contains datablock(s) I, global. DOI: 10.1107/S1600536813017248/qm2095sup1.cif

Structure factors: contains datablock(s) I. DOI: 10.1107/S1600536813017248/qm2095Isup2.hkl

Supplementary material file. DOI: 10.1107/S1600536813017248/qm2095Isup3.cml

Additional supplementary materials:  crystallographic information; 3D view; checkCIF report

## Figures and Tables

**Table 1:** Hydrogen-bond geometry (Å, °)

*D*—H⋯*A*	*D*—H	H⋯*A*	*D*⋯*A*	*D*—H⋯*A*
N1—H10⋯O3^i^	0.91 (2)	2.17 (2)	3.065 (2)	168.7 (18)
N1—H11⋯O2^ii^	0.89 (2)	2.02 (3)	2.912 (2)	177 (2)

Symmetry codes: (i) 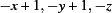; (ii) 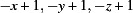.

**Fig. 1.**
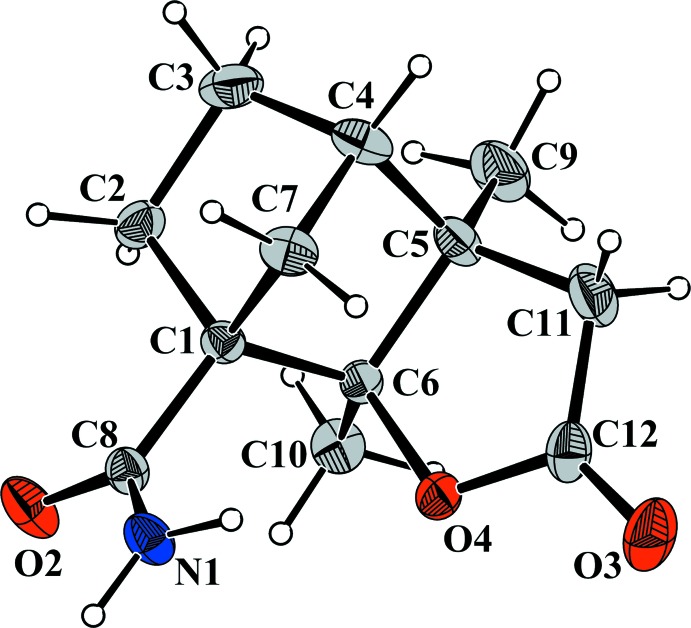
The molecular structure and atom nunbering scheme for the title compound, showing 30% probability displacement ellipsoids.

**Fig. 2.**
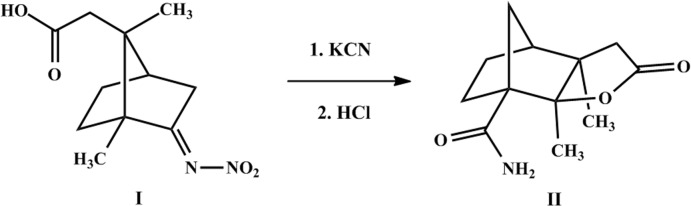
The synthetic route to the title compound (II).

## supplementary crystallographic information

### Comment

Nitroimines and their derivatives have found a numerous applications in organic synthesis (Squire *et al.*, 2002; Bulman Page *et al.*, 2000; Lalk *et al.*, 1999), as organocatalysis (Parrott, R. W. *et al.*, 2008) and in medicinal chemistry (Ranise *et al.*, 1990; Bondavalli, *et al.*, 1987). Herein, we report synthesis and crystal structure of the title compound (II), the novel 3,7-dimethyl-3-oxohexahydro-4,7-methano-2-benzofuran-4(1*H*)-carboxamide , C_12_H_17_NO_3_ (Fig. 1)obtained *via* selective Wagner-Meerwein type rearrangement of potassium cyanide adducts of 1,7-dimethyl-2-(nitroimino)bicyclo[2.2.1]hept-7-ylacetic acid (I) (see Fig. 2).

In the structure of (II) (Fig. 1), angle deviations at Csp^3^ atoms range from 94.22 to 118.10 (16) °. Thus the C1—C7—C4 angle as in other previously reported compounds has a reduced value of 94.22 (12) ° (Noe *et al.*, 1996; Knollmuller *et al.*, 1998). Most bond distances for compound (I) were in the expected range however, the C5-C6 bond length is 1.573 (2) Å, which is apparently due to steric repulsion between the methyl groups on these atoms, this also leads to an increase in the angles C5C6C10 and C6C5C9 to 118.10 (13) and 115.04 (15) °, respectively. It is interesting to note that the bond O4—C6 is slightly longer (1.4714 (16) Å) and O4—C12 shorter (1.335 (2) Å) compared with the values previously found (average 1.45 and 1.36 Å, respectively). Intermolecular hydrogen bonds are formed through N—H···O—C and N—H···O═C between the amide and two O-atom acceptors of lactone group. (Table 1).

### Experimental

The synthesis of the nitroimine (I) (Fig. 2) was carried out as follows. A solution of compound I (1.00 g, 4.16 mmol) in methanol (10 ml) was added to a mixture of acetone cyanohydrin 2 mmol (0.708 g) and potassium hydroxide 1.5 mmol (0.35 g) in distilled water. The resulting mixture was stirred and refluxed for 20 min. After cooling in ice an excess of 3 N aqueous hydrochloric acid was added over 5 min with vigorous stirring. Almost immediate precipitation of carboxamide was accompanied by gas (N_2_O) evolution. The amide was filtered off, washed with distilled water (2x10 mL), dried in a vacuum desiccator overnight and recrystallized from absolute 2-propanol. Yield: 0.86 g 93%; m.p.: 253 °C. 1H NMR (400 MHz, [D6]DMSO, TMS, δ): 1.201 (s, 3 H), 1.516 (s, 3 H), 1.531–1.577 (m, 1 H), 1.630–1.95 (d, J=11.1Hz, 1H), 1.693–1.794 (m, 3 H), 1.952 (d, J=11.1, 1 H), 2.065 (bs, 1 H), 2.601 (s, 2 H); 13C{1H} NMR (100.70 MHz, [D6]DMSO, TMS, δ): 16.55, 20.76, 24.99, 28.53, 37.35, 44.82, 48.14, 49.56, 62.19, 97.18, 177.35,178.65; (KBr plates, cm -1): 3421.23, 3157.94, 1755.23, 1678.33.

### Refinement

Amide H-atoms were located in a difference-Fourier synthesis and both positional and displacement parameters were allowed to refine. Other hydrogen atoms were positioned geometrically, with C—H = 0.96–0.98 Å and were allowed to ride on their parent atoms, with *U*_iso_(H) = 1.2*U*_eq_(methine or methylene C) or 1.5*U*_eq_(methyl C). In the absence of a suitable heavy atom, the absolute configuration of the title compound could not be determined (1146 Friedel pairs).

### Crystal data

**Table:**

C_12_H_17_NO_3_	*F*(000) = 240
*M**_r_* = 223.27	*D*_x_ = 1.332 Mg m^−^^3^
Triclinic, *P*1	Melting point: 531 K
*a* = 7.0659 (3) Å	Mo *K*α radiation, λ = 0.71073 Å
*b* = 7.8206 (3) Å	Cell parameters from 8729 reflections
*c* = 10.4595 (3) Å	θ = 2.0–27.0°
α = 79.667 (2)°	µ = 0.10 mm^−^^1^
β = 80.471 (2)°	*T* = 296 K
γ = 81.579 (2)°	Block, colourless
*V* = 556.69 (4) Å^3^	0.25 × 0.2 × 0.15 mm
*Z* = 2	

### Data collection

**Table:**

Bruker SMART APEXII CCD area-detector diffractometer	2405 independent reflections
Radiation source: fine-focus sealed tube	1754 reflections with *I* > 2σ(*I*)
Graphite monochromator	*R*_int_ = 0.037
ω scans	θ_max_ = 27.0°, θ_min_ = 2.0°
Absorption correction: multi-scan (*SADABS*; Bruker, 2008)	*h* = −9→6
*T*_min_ = 0.977, *T*_max_ = 0.986	*k* = −9→9
8729 measured reflections	*l* = −13→12

### Refinement

**Table:**

Refinement on *F*^2^	Primary atom site location: structure-invariant direct methods
Least-squares matrix: full	Secondary atom site location: difference Fourier map
*R*[*F*^2^ > 2σ(*F*^2^)] = 0.044	Hydrogen site location: inferred from neighbouring sites
*wR*(*F*^2^) = 0.141	H atoms treated by a mixture of independent and constrained refinement
*S* = 0.92	*w* = 1/[σ^2^(*F*_o_^2^) + (0.1*P*)^2^] where *P* = (*F*_o_^2^ + 2*F*_c_^2^)/3
2405 reflections	(Δ/σ)_max_ = 0.001
213 parameters	Δρ_max_ = 0.17 e Å^−^^3^
0 restraints	Δρ_min_ = −0.21 e Å^−^^3^

### Special details

**Table:**

Geometry. All esds (except the esd in the dihedral angle between two l.s. planes) are estimated using the full covariance matrix. The cell esds are taken into account individually in the estimation of esds in distances, angles and torsion angles; correlations between esds in cell parameters are only used when they are defined by crystal symmetry. An approximate (isotropic) treatment of cell esds is used for estimating esds involving l.s. planes.
Refinement. Refinement of F^2^ against ALL reflections. The weighted R-factor wR and goodness of fit S are based on F^2^, conventional R-factors R are based on F, with F set to zero for negative F^2^. The threshold expression of F^2^ > 2sigma(F^2^) is used only for calculating R-factors(gt) etc. and is not relevant to the choice of reflections for refinement. R-factors based on F^2^ are statistically about twice as large as those based on F, and R- factors based on ALL data will be even larger.

### Fractional atomic coordinates and isotropic or equivalent isotropic displacement parameters (Å^2^)

**Table:**

	*x*	*y*	*z*	*U*_iso_*/*U*_eq_	
N1	0.3999 (2)	0.54171 (18)	0.34083 (15)	0.0369 (4)	
O2	0.33949 (19)	0.33578 (16)	0.51638 (12)	0.0494 (4)	
O3	0.5612 (2)	0.31087 (19)	−0.04693 (13)	0.0603 (4)	
O4	0.45280 (15)	0.25378 (14)	0.16493 (10)	0.0347 (3)	
C1	0.1850 (2)	0.32889 (19)	0.33196 (14)	0.0273 (4)	
C2	0.0143 (2)	0.2540 (2)	0.42730 (17)	0.0375 (4)	
C3	−0.1362 (3)	0.2492 (3)	0.3377 (2)	0.0484 (5)	
C4	−0.0303 (2)	0.3074 (2)	0.20074 (19)	0.0429 (4)	
C5	0.1373 (2)	0.1653 (2)	0.16319 (15)	0.0358 (4)	
C6	0.2885 (2)	0.18268 (18)	0.25359 (14)	0.0269 (3)	
C7	0.0779 (3)	0.4509 (2)	0.22716 (19)	0.0381 (4)	
C8	0.3163 (2)	0.4028 (2)	0.40296 (15)	0.0298 (4)	
C9	0.0736 (4)	−0.0173 (3)	0.1799 (2)	0.0540 (5)	
C10	0.3770 (3)	0.0174 (2)	0.33196 (19)	0.0393 (4)	
C11	0.2432 (3)	0.2168 (3)	0.02428 (19)	0.0523 (5)	
C12	0.4339 (3)	0.2662 (2)	0.03857 (16)	0.0404 (4)	
H4	−0.113 (3)	0.340 (3)	0.133 (2)	0.060 (6)*	
H21	0.057 (2)	0.134 (2)	0.4786 (17)	0.039 (5)*	
H22	−0.035 (3)	0.332 (2)	0.4956 (19)	0.048 (5)*	
H31	−0.255 (3)	0.336 (3)	0.358 (2)	0.064 (6)*	
H32	−0.182 (3)	0.137 (3)	0.347 (2)	0.059 (6)*	
H71	−0.003 (3)	0.545 (3)	0.2658 (18)	0.051 (5)*	
H72	0.165 (2)	0.501 (2)	0.1515 (17)	0.035 (5)*	
H91	0.027 (3)	−0.063 (3)	0.274 (2)	0.063 (6)*	
H92	−0.029 (4)	−0.010 (4)	0.128 (3)	0.090 (8)*	
H93	0.177 (3)	−0.102 (3)	0.143 (2)	0.068 (7)*	
H101	0.470 (4)	0.049 (3)	0.379 (2)	0.070 (7)*	
H102	0.277 (3)	−0.046 (3)	0.391 (2)	0.055 (6)*	
H103	0.446 (3)	−0.060 (3)	0.270 (2)	0.063 (6)*	
H110	0.171 (4)	0.314 (3)	−0.025 (2)	0.082 (8)*	
H111	0.267 (3)	0.131 (3)	−0.027 (3)	0.078 (8)*	
H10	0.397 (3)	0.577 (3)	0.253 (2)	0.054 (6)*	
H11	0.479 (3)	0.583 (3)	0.383 (2)	0.059 (6)*	

### Atomic displacement parameters (Å^2^)

**Table:**

	*U*^11^	*U*^22^	*U*^33^	*U*^12^	*U*^13^	*U*^23^
N1	0.0469 (8)	0.0356 (8)	0.0328 (8)	−0.0195 (6)	−0.0102 (7)	−0.0014 (6)
O2	0.0690 (9)	0.0520 (8)	0.0344 (7)	−0.0301 (6)	−0.0203 (6)	0.0048 (6)
O3	0.0725 (9)	0.0650 (9)	0.0380 (8)	−0.0219 (7)	0.0160 (7)	−0.0052 (6)
O4	0.0331 (6)	0.0417 (6)	0.0298 (6)	−0.0112 (5)	0.0009 (5)	−0.0062 (5)
C1	0.0274 (7)	0.0269 (7)	0.0283 (8)	−0.0067 (6)	−0.0041 (6)	−0.0032 (6)
C2	0.0325 (8)	0.0410 (10)	0.0392 (10)	−0.0121 (7)	0.0039 (7)	−0.0087 (8)
C3	0.0284 (9)	0.0539 (12)	0.0643 (13)	−0.0113 (8)	−0.0026 (8)	−0.0118 (10)
C4	0.0378 (9)	0.0455 (10)	0.0491 (11)	−0.0073 (7)	−0.0221 (8)	−0.0013 (8)
C5	0.0419 (9)	0.0398 (9)	0.0309 (9)	−0.0144 (7)	−0.0121 (7)	−0.0051 (7)
C6	0.0284 (7)	0.0282 (7)	0.0244 (7)	−0.0103 (6)	−0.0010 (6)	−0.0019 (6)
C7	0.0376 (9)	0.0300 (8)	0.0464 (10)	−0.0018 (7)	−0.0136 (8)	−0.0005 (8)
C8	0.0334 (8)	0.0284 (7)	0.0291 (8)	−0.0060 (6)	−0.0049 (6)	−0.0066 (6)
C9	0.0637 (13)	0.0500 (12)	0.0584 (14)	−0.0241 (10)	−0.0141 (11)	−0.0167 (10)
C10	0.0441 (10)	0.0319 (9)	0.0406 (10)	−0.0016 (7)	−0.0089 (8)	−0.0016 (7)
C11	0.0651 (13)	0.0670 (14)	0.0298 (10)	−0.0192 (11)	−0.0124 (9)	−0.0069 (9)
C12	0.0536 (10)	0.0378 (9)	0.0277 (9)	−0.0099 (8)	0.0020 (8)	−0.0034 (7)

### Geometric parameters (Å, º)

**Table:**

N1—C8	1.326 (2)	C4—C5	1.552 (2)
N1—H10	0.91 (2)	C4—H4	0.97 (2)
N1—H11	0.89 (2)	C5—C9	1.531 (2)
O2—C8	1.2349 (19)	C5—C11	1.533 (3)
O3—C12	1.202 (2)	C5—C6	1.573 (2)
O4—C12	1.335 (2)	C6—C10	1.513 (2)
O4—C6	1.4714 (16)	C7—H71	0.97 (2)
C1—C8	1.512 (2)	C7—H72	0.979 (16)
C1—C7	1.540 (2)	C9—H91	1.00 (2)
C1—C2	1.546 (2)	C9—H92	0.97 (3)
C1—C6	1.551 (2)	C9—H93	0.99 (2)
C2—C3	1.538 (3)	C10—H101	0.97 (3)
C2—H21	1.027 (18)	C10—H102	0.99 (2)
C2—H22	1.01 (2)	C10—H103	0.98 (2)
C3—C4	1.526 (3)	C11—C12	1.492 (3)
C3—H31	1.02 (2)	C11—H110	0.96 (3)
C3—H32	0.96 (2)	C11—H111	0.91 (3)
C4—C7	1.534 (2)		
			
C8—N1—H10	120.3 (13)	O4—C6—C1	107.26 (11)
C8—N1—H11	117.4 (14)	C10—C6—C1	116.77 (13)
H10—N1—H11	121.0 (19)	O4—C6—C5	106.01 (11)
C12—O4—C6	112.40 (12)	C10—C6—C5	118.10 (13)
C8—C1—C7	119.50 (13)	C1—C6—C5	103.22 (11)
C8—C1—C2	111.80 (12)	C4—C7—C1	94.22 (12)
C7—C1—C2	101.23 (13)	C4—C7—H71	115.3 (12)
C8—C1—C6	114.09 (12)	C1—C7—H71	109.9 (12)
C7—C1—C6	101.17 (12)	C4—C7—H72	114.6 (10)
C2—C1—C6	107.58 (12)	C1—C7—H72	113.0 (10)
C3—C2—C1	103.89 (13)	H71—C7—H72	109.2 (15)
C3—C2—H21	113.5 (10)	O2—C8—N1	122.17 (15)
C1—C2—H21	111.5 (9)	O2—C8—C1	119.81 (13)
C3—C2—H22	112.5 (11)	N1—C8—C1	118.00 (14)
C1—C2—H22	109.8 (10)	C5—C9—H91	111.8 (13)
H21—C2—H22	105.7 (14)	C5—C9—H92	108.2 (16)
C4—C3—C2	102.89 (13)	H91—C9—H92	109.9 (18)
C4—C3—H31	110.1 (12)	C5—C9—H93	112.0 (13)
C2—C3—H31	110.3 (13)	H91—C9—H93	110.3 (18)
C4—C3—H32	113.3 (13)	H92—C9—H93	104 (2)
C2—C3—H32	114.1 (13)	C6—C10—H101	108.3 (13)
H31—C3—H32	106.1 (17)	C6—C10—H102	111.6 (11)
C3—C4—C7	101.05 (15)	H101—C10—H102	112.2 (18)
C3—C4—C5	110.33 (14)	C6—C10—H103	108.2 (12)
C7—C4—C5	102.39 (13)	H101—C10—H103	108.1 (18)
C3—C4—H4	114.4 (11)	H102—C10—H103	108.2 (17)
C7—C4—H4	117.0 (12)	C12—C11—C5	107.10 (15)
C5—C4—H4	110.7 (12)	C12—C11—H110	109.9 (15)
C9—C5—C11	110.84 (16)	C5—C11—H110	111.7 (14)
C9—C5—C4	113.29 (15)	C12—C11—H111	107.6 (16)
C11—C5—C4	111.81 (16)	C5—C11—H111	115.3 (16)
C9—C5—C6	115.04 (15)	H110—C11—H111	105 (2)
C11—C5—C6	103.15 (13)	O3—C12—O4	120.98 (17)
C4—C5—C6	102.04 (12)	O3—C12—C11	127.97 (17)
O4—C6—C10	104.66 (12)	O4—C12—C11	111.04 (14)
			
C8—C1—C2—C3	160.01 (14)	C4—C5—C6—O4	−111.46 (12)
C7—C1—C2—C3	31.68 (16)	C9—C5—C6—C10	8.6 (2)
C6—C1—C2—C3	−73.98 (16)	C11—C5—C6—C10	−112.21 (17)
C1—C2—C3—C4	4.31 (18)	C4—C5—C6—C10	131.69 (15)
C2—C3—C4—C7	−39.09 (17)	C9—C5—C6—C1	−121.93 (16)
C2—C3—C4—C5	68.70 (18)	C11—C5—C6—C1	117.25 (14)
C3—C4—C5—C9	52.1 (2)	C4—C5—C6—C1	1.15 (14)
C7—C4—C5—C9	159.00 (16)	C3—C4—C7—C1	57.55 (14)
C3—C4—C5—C11	178.22 (15)	C5—C4—C7—C1	−56.36 (15)
C7—C4—C5—C11	−74.89 (17)	C8—C1—C7—C4	−177.35 (13)
C3—C4—C5—C6	−72.16 (15)	C2—C1—C7—C4	−54.16 (15)
C7—C4—C5—C6	34.74 (15)	C6—C1—C7—C4	56.51 (14)
C12—O4—C6—C10	119.82 (15)	C7—C1—C8—O2	150.48 (16)
C12—O4—C6—C1	−115.51 (14)	C2—C1—C8—O2	32.62 (19)
C12—O4—C6—C5	−5.73 (16)	C6—C1—C8—O2	−89.74 (17)
C8—C1—C6—O4	−54.29 (15)	C7—C1—C8—N1	−28.0 (2)
C7—C1—C6—O4	75.35 (13)	C2—C1—C8—N1	−145.89 (14)
C2—C1—C6—O4	−178.94 (11)	C6—C1—C8—N1	91.76 (16)
C8—C1—C6—C10	62.68 (18)	C9—C5—C11—C12	−125.98 (18)
C7—C1—C6—C10	−167.67 (14)	C4—C5—C11—C12	106.57 (18)
C2—C1—C6—C10	−61.96 (18)	C6—C5—C11—C12	−2.3 (2)
C8—C1—C6—C5	−165.99 (12)	C6—O4—C12—O3	−175.02 (14)
C7—C1—C6—C5	−36.34 (13)	C6—O4—C12—C11	4.3 (2)
C2—C1—C6—C5	69.37 (14)	C5—C11—C12—O3	178.28 (17)
C9—C5—C6—O4	125.45 (15)	C5—C11—C12—O4	−1.0 (2)
C11—C5—C6—O4	4.64 (16)		

### Hydrogen-bond geometry (Å, º)

**Table:**

*D*—H···*A*	*D*—H	H···*A*	*D*···*A*	*D*—H···*A*
N1—H10···O3^i^	0.91 (2)	2.17 (2)	3.065 (2)	168.7 (18)
N1—H11···O2^ii^	0.89 (2)	2.02 (3)	2.912 (2)	177 (2)

Symmetry codes: (i) −*x*+1, −*y*+1, −*z*; (ii) −*x*+1, −*y*+1, −*z*+1.
